# Clinical - epidemiological aspects and diagnosis of an outbreak of anthrax in Moldova

**DOI:** 10.1186/1471-2334-13-S1-P72

**Published:** 2013-12-16

**Authors:** Valentin Cebotarescu, Victor Pântea, Gheorghe Plăcintă, Tiberiu Holban, Lilia Cojuhari, Ludmila Simonov, Stela Semeniuc, Galina Chiriacov, Nicolai Petuhov, Lucia Moraru, Tatiana Musteață, Valentina Smeşnoi

**Affiliations:** 1 Department of Infectious Diseases, Faculty for Continuing Medical Education, Nicolae Testemițanu State Medical and Pharmacy University, Chişinău, Republic of Moldova; 2 Department of Infectious Diseases, Tropical and Medical Parazitology, Nicolae Testemițanu State Medical and Pharmaceutical University, Chişinău, Republic of Moldova; 3 Toma Ciorbă Clinical Hospital for Infectious Diseases, Chişinău, Republic of Moldova; 4 Chiril Draganiuc Institute of Phthisiopneumology, Chişinău, Republic of Moldova

## Background

Anthrax is an acute disease that affects both humans and animals. Most forms of the disease are lethal. Anthrax commonly infects herbivorous mammals that ingest or inhale the spores while grazing. Diseased animals can spread anthrax to humans, either by direct contact or by meat consumption.

## Methods

We report a series of 4 cases of patients diagnosed with anthrax who have become infected at the slaughter house or from infected cattle. The diagnosis was confirmed by molecular biological testing with the identification of *B anthracis* DNA in carbuncular exudate.

## Results

The mean age of the patients was 30, and they were admitted in the Clinical Hospital for Infectious Diseases “Toma Ciorbă” in July, 2013. The source of infection was represented by cattle, in which the infection was confirmed by bacteriological method. The infection occurred through contact with sick animals during slaughter. The disease was developed after an incubation period, which lasted on average 9 days. The first two patients were admitted on the fourth day from the onset of symptoms, one on the third and the other on the second day following onset.

All the patients were diagnosed with anthrax, a cutaneous form, carbuncles, evolving moderately in 3 patients. One patient had a severe adverse development of encephalopathy and edema as a result of practicing thoracic incisions around carbuncle in a surgical ward (before determination of anthrax diagnosis). The clinical picture consisted of the presence of general symptoms: fever, fatigue, headache and skin lesions: papule, vesicle and then rapidly evolving into ulceration, carbuncles formation covered with a black crust surrounded by pruritic edema, painless. Carbuncles location was on the upper limbs of the three patients and one in the cervical region. Antibacterial treatment with penicillin G and cephalosporin was effective in all patients requiring hospitalization for an average of 25 days.

## Conclusion

In all the patients the disease evolved in cutaneous forms, moderate (3) and severe (1). The diagnosis was confirmed by molecular biological method for identifying *B anthracis* DNA in the carbuncular exudate. Treatment was complex: antibacterial, pathogenic and symptomatic.

